# Innate and adaptive immune-directed tumour microenvironment in pancreatic ductal adenocarcinoma

**DOI:** 10.3389/fimmu.2024.1323198

**Published:** 2024-02-07

**Authors:** Ann Mary Joseph, Ahmad Al Aiyan, Basel Al-Ramadi, Shiv K. Singh, Uday Kishore

**Affiliations:** ^1^ Department of Veterinary Medicine (CAVM), United Arab Emirates University, Al Ain, United Arab Emirates; ^2^ Department of Medical Microbiology and Immunology, College of Medicine and Health Sciences, United Arab Emirates University, Al Ain, United Arab Emirates; ^3^ Zayed Center for Health Sciences, United Arab Emirates University, Al Ain, United Arab Emirates; ^4^ ASPIRE Precision Medicine Research Institute Abu Dhabi, United Arab Emirates University, Al Ain, United Arab Emirates; ^5^ Department of Gastroenterology and Gastrointestinal Oncology, University Medical Center, Goettingen, Germany

**Keywords:** PDAC, TME, immune surveillance, immune suppression, EMT, macrophages, TNF-α

## Abstract

One of the most deadly and aggressive cancers in the world, pancreatic ductal adenocarcinoma (PDAC), typically manifests at an advanced stage. PDAC is becoming more common, and by the year 2030, it is expected to overtake lung cancer as the second greatest cause of cancer-related death. The poor prognosis can be attributed to a number of factors, including difficulties in early identification, a poor probability of curative radical resection, limited response to chemotherapy and radiotherapy, and its immunotherapy resistance. Furthermore, an extensive desmoplastic stroma that surrounds PDAC forms a mechanical barrier that prevents vascularization and promotes poor immune cell penetration. Phenotypic heterogeneity, drug resistance, and immunosuppressive tumor microenvironment are the main causes of PDAC aggressiveness. There is a complex and dynamic interaction between tumor cells in PDAC with stromal cells within the tumour immune microenvironment. The immune suppressive microenvironment that promotes PDAC aggressiveness is contributed by a range of cellular and humoral factors, which itself are modulated by the cancer. In this review, we describe the role of innate and adaptive immune cells, complex tumor microenvironment in PDAC, humoral factors, innate immune-mediated therapeutic advances, and recent clinical trials in PDAC.

## 1 Introduction

Pancreatic ductal adenocarcinoma (PDAC) is one of the deadliest solid tumours in humans. It is the most frequent form of pancreatic cancer, 90% of all pancreas neoplasms, which is characterised by tubular adenocarcinoma of the ductal glands (1, 2). Pancreatic cancer and pancreatic ductal adenocarcinoma are sometimes used interchangeably. Only 11% of patients with PDAC survive for at least 5 years (3). Over 400,000 people die from PDAC every year, the seventh most common cancer-related cause of death worldwide (4). A usually poor prognosis is projected for the more than 450,000 patients who receive annual diagnosis (5). It is predicted that pancreatic cancer-related death will overtake lung cancer as the second most prevalent cause of cancer-related death in the United States by 2030 (6). Along with the aggressive tumour biology, the pancreas’ central placement within the abdominal cavity, the lack of a distinct organ capsule, and the abundance of nearby blood and lymphatic arteries all contribute to the tumor’s ability to spread locally and elsewhere such as liver, lung, bone and brain (7). The pancreas, a comparatively clean organ, with very few lymphocytes, is located in the retroperitoneum and has no direct contact with the outside world; instead, it communicates with the digestive tract solely through the pancreatic duct. Therefore, very few lymphocytes can be seen in healthy pancreatic tissue (8, 9). In contrast to other malignancies, the incidence of pancreatic cancer is still rising while survival rates are barely improving.

A few recent reviews in the field describe the immunosuppressive TiME in PDAC and the TME targeted therapeutic approaches (10), pro- and anti-tumour properties of immune cells (11), the effector immune cells with potential biomarkers and targets (12), and the need for reprogramming of the stroma for the development of new therapeutic strategies (13). In this review, we have examined the immune landscape in human PDAC more holistically and how that affects survival and treatment for PDAC patients. This review also includes some of the important areas such as humoral immune factors, its significance, and the coexistence of classical and basal-like phenotypes.

### 1.1 Therapy

The main therapeutic modalities for PDAC are surgical resection, chemotherapy, and radiotherapy. In most cases, the only form of treatment that has a chance of being curative is radical surgical resection (14, 15). Less than 25–30% of all PDAC patients are considered candidates for partial pancreatectomy at the time of diagnosis (16). Nearly 80% of PDAC patients cannot have a curative resection due to the stromal microenvironment which plays a role in malignant transformation, local invasion, and distant metastasis (17, 18). The development of immune checkpoint blockade (ICB) therapy has revolutionized cancer treatment, but the PDAC immunotherapy regimen, whether used alone or in combination with chemotherapy, has not shown encouraging results in patients with metastatic PDAC (mPDAC) (19). Borderline resectable or locally advanced PDAC patients, have significantly better survival rates in those patients who received neoadjuvant therapy (20, 21). The neoadjuvant therapy regimen includes chemotherapy with 5-fluorouracil (FOLFIRINOX/FOLFOX), gemcitabine (gemcitabine/nab-paclitaxel) or chemoradiotherapy before surgery. The CA19-9 level is considered a specific biomarker for tumor resectability and overall survival (22).

Conventional cytotoxic therapies such as chemotherapy and radiation therapy have not increased the chances of survival for patients with pancreatic cancer. Since 2011, 5-fluorouracil/leucovorin with irinotecan and oxaliplatin (FOLFIRINOX) and nab-paclitaxel with gemcitabine have been the preferred treatments for mPDAC. Response rates for these treatments range between 23% and 31%, progression-free survival time ranges from 5.5 to 6.6 months, and overall survival times range from 8.5 to 11 months. The only targeted treatment for PDAC that the US Food and Drug Administration (FDA) has approved is erlotinib, an epidermal growth factor receptor (EGFR) inhibitor, in combination with gemcitabine hydrochloride in patients with metastatic, locally advanced, or unresectable PDAC. The absolute benefit of gemcitabine and erlotinib, however, is also negligible for up to 2 weeks (23).

**Figure 1:**
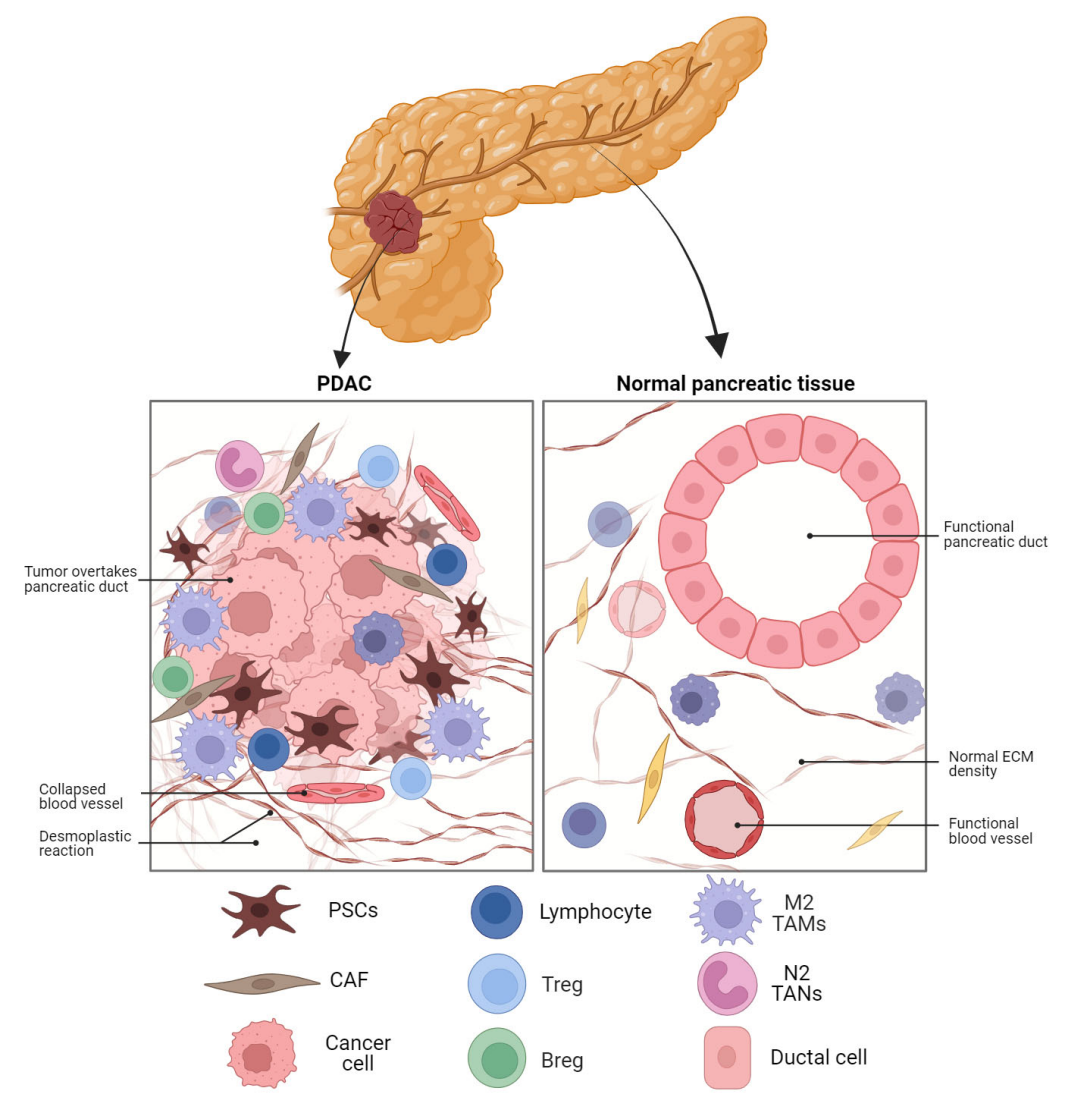
Schematic representation of the PDAC TME compared to normal pancreatic tissue. Fibroblast activated in the tissue to CAFs are the dominant cell type in PDAC along with M2 TAMs and MDSC. The dense desmoplastic reaction and collapsed blood vessels provide barriers to cytotoxic T cell infiltration. PDAC, pancreatic ductal adenocarcinoma; ECM, extracellular matrix; PSC, pancreatic stellate cell; CAF, cancer-associated fibroblast;Treg, regulatory T cell; Breg, regulatory B cell; TAM, tumour-associated macrophage; TAN, tumour-associated neutrophil.

Despite advancements in pancreatic cancer research, screening, and treatment strategies, PDAC has a poor prognosis and resistance to many treatments, including immunotherapy (24). A large matrix of stromal cells is strongly connected with the poor prognosis of PDAC (25). PDAC is characterised by a desmoplastic stroma, a fibrotic TME with respect to normal pancreatic tissue as illustrated in **Figure 1**. Additionally, different epigenetic modifications as well as mutations in protooncogenes and tumour suppressor genes are seen in the stromal cells surrounding the tumour as well as the tumour epithelium (26, 27). The compact dysplastic stroma of PDAC is a significant barrier to chemotherapeutic agents. Thus, stroma-targeting therapy has been recognised as a prospective approach to enhance the effectiveness of chemotherapy, and hence, patient survival rates (28).

A major component of the stroma in PDAC, hyaluronic acid (HA), interacts with cell surface receptors CD44 and receptor for HA-mediated motility (RHAMM) to promote tumour cell survival and to initiate signalling pathways associated with tumour cell proliferation, migration, and invasion (29–32). Hence, the targeting of HA is regarded as a promising therapeutic approach in the context of PDAC. PEGylated hyaluronidase (PEGPH20) refers to a PEGylated nanoscale complex that consists of recombinant human hyaluronidase (33, 34). Several studies have demonstrated that PEGPH20 has the ability to degrade HA, remodel tumour vasculature, and enhance the effectiveness of chemotherapeutic drugs (33, 35, 36). The study HALO-109-202, a phase II clinical trial, examined the effects of combining PEGPH20 with Abraxane (an albumin-bound paclitaxel nanocomplex) and gemcitabine in 279 patients diagnosed with mPDAC. The results demonstrated a significant increase in progression-free survival and overall survival among patients with elevated levels of HA (37). However, the phase III clinical study failed to considerably improve the PDAC patients’ overall survival.

Collagen represents another significant constituent within the extracellular matrix (ECM) of tumours. High levels of fibrillar collagens found in the stroma of PDAC play a critical role in promoting tumour cell survival and tumour progression. This process is mediated by the involvement of discoidin domain receptor 1 and 2 (DDR1 and DDR2). Huo et al. revealed that there was a significant correlation between elevated expression levels of DDR1 and an increased risk of unfavourable prognosis in PDAC patients (38). A small molecule inhibitor targeting DDR1 resulted in a decrease in fibrillar collagen deposition and an enhancement in the efficacy of chemotherapy in orthotopic mouse models of PDAC (39). KI-301690, a small molecule that disrupts DDR1 signaling, is a selective DDR1 inhibitor. A combination treatment with gemcitabine significantly inhibited the growth of pancreatic cancer cells (40).

Hedgehog (Hh) signalling pathway is typically characterised by an increased activity in PDAC via the activation of pancreatic stellate cells (41). This pathway has been shown to play a role in the regulation of stroma deposition (42). Multiple strategies have been developed with the aim of treating PDAC through the inhibition of the Hh signalling pathway, with the ultimate goal of eradicating the tumour stroma (43). Cyclopamine, a steroidal alkaloid of natural origin, has been found to effectively inhibit the Hh signalling pathway by binding to the Smoothened (SMO) protein (44). In the PDAC xenograft mouse model, it was found that the fibronectin content was decreased and tumour vascularization was increased. Co-administration of cyclopamine with paclitaxel-loaded nanoparticles resulted in a significant enhancement of tumour growth inhibition (45). The anti-tumor efficacy was mediated by increased tumor infiltration of CD8^+^ T cells without concomitant infiltration of immune suppressive cells, and by the coordinated action of Paclitaxel and IFN-γ (46). Another polymeric conjugate of docetaxel and cyclopamine has been examined for its anti-cancer effect in murine PDAC (47). This combination therapy resulted in greater inhibition of orthotopic pancreatic tumor growth.

The majority of PDAC patients have non-resectable tumours by the time they develop symptoms such as weight loss, abdominal pain and jaundice (48). Early detection of PDAC improves survival rates, but its low prevalence makes screening the general population impractical. Screening subgroups may include people with germline mutations, pancreatitis, mucinous pancreatic cysts, and elderly new-onset diabetics (49). For accurate diagnosis, high-resolution ultrasound, endoscopic ultrasound (EUS), computed tomography (CT), and magnetic resonance imaging (MRI) are needed.

### 1.2 Biomarkers and oncogenic mutations

Advanced PDAC has few treatment options, making early detection crucial for prognosis. Thus, developing diagnostic biomarkers for high-risk populations is important. CA19-9, the only FDA-approved biomarker for the diagnosis and monitoring of PDAC, is probably the most extensively validated biomarker that has diagnostic, prognostic and surveillance value (50, 51). CA125, CA72-4, CA50, CA199, and CA242 are other antigens used as biomarkers (52, 53). A single diagnostic potential for any of these biomarkers could not be established; however, when used along with CA 19-9, they may help distinguish between benign and malignant pancreatic lesions. Similarly, CA19-9, when combined with CEA, appears to have a better prognostic value, particularly in advanced PDAC (54).

Typically, PDAC is characterised by the presence of oncogenic mutations in genes such as KRAS and loss-of-function mutations in tumour suppressors such as TP53, CDNK2A, SMAD4, and BRCA2. These biomarkers and genomic mutations have the potential to function as targets or prognostic indicators, depending on the expression. PDAC originates from a series of precursor lesions, such as pancreatic intraepithelial neoplasia (PanIN), intraductal papillary mucinous neoplasm (IPMN), and mucinous cystic neoplasm (MCN) (55). In most cases, KRAS mutations emerge in PanIN-1 lesions and drive the initiation process, while CDKN2A mutations emerge in PanIN-2 and drive the disease forward. Mutations in TP53 and SMAD4, are frequently found in PanIN-3 and invasive tumours (56, 57). Approximately 95% of pancreatic tumours exhibit RAS mutations, with KRAS alterations being the most prevalent, accounting for 85% of cases. Additionally, KRAS stimulates the nuclear factor κB (NF-κB) pathway, which is linked to the development of a strong inflammatory response (58). Besides mutations in KRAS, inactivation of CDKN2A is observed in approximately 90% of PDAC cases, while SMAD4/DPC4 alterations are present in approximately 55% of cases (59). Also, a significant proportion of PDAC cases, ranging from approximately 50% to 70%, exhibit mutations in the TP53 gene (60). The SMAD4 gene is deactivated in approximately 60% of cases of PDAC (61). This gene plays a crucial role as an effector in the transforming growth factor β (TGF-β) signalling, which is also disrupted in 47% of PDAC cases (62, 63). Dysregulation of various critical processes-related signalling pathways, such as apoptosis and cell proliferation, occurs because of these mutations.

### 1.3 Classification of PDAC subtypes

**Figure 2:**
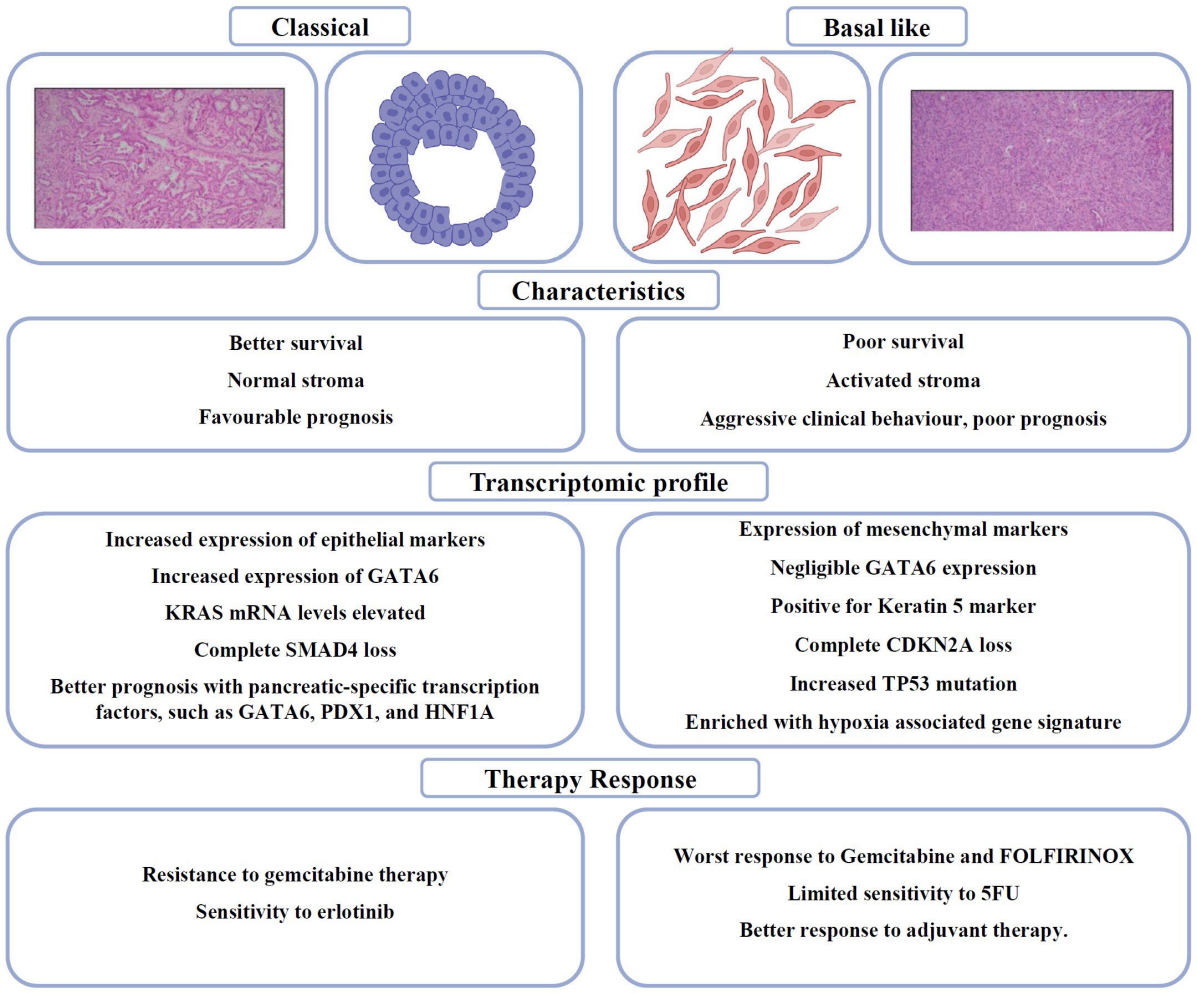
Schematic and H&E sections PDAC (Scale: 200 μm) to distinguish between Classical and Basal like subtypes in PDAC (64), summarising the clinicopathological differences, genetic signatures and therapy responses.

Genomic profiling at a large scale has shown that PDAC has two different histological types: “classical” and “basal-like”. As shown in **Figure 2**, the “Classical” or progenitor subtype was distinguished by the expression of epithelial markers and a good prognosis, while the “Basal-like,” squamous or quasi-mesenchymal subtype was characterised by the expression of mesenchymal markers and aggressive/metastatic properties. There is still disagreement over how to actually use the subtype classification for clinical decision-making in PDAC, despite the fact that these molecular subtypes of PDAC may offer new avenues for precision medicine approaches (65). Collisson et al. conducted transcriptome analyses on tissue samples of PDAC, as well as human and murine PDAC cell lines, and identified three distinct molecular subtypes of PDAC, namely the classical, quasi-mesenchymal, and exocrine-like subtypes (66). The classical subtype is distinguished by the activation of genes associated with epithelial and adhesion functions. In contrast, the quasi-mesenchymal subtype predominantly exhibits the expression of genes related to mesenchymal characteristics. Additionally, the exocrine-like subtype is characterised by the upregulation of genes associated with digestive enzymes. It is noteworthy that these subtypes exhibit relevance in terms of survival, as the classical subtype is associated with the most favorable prognosis, while the quasi-mesenchymal subtype is linked with the poorest prognosis (66). Moreover, it has been observed that PDAC cell lines belonging to the classical subtype exhibit resistance to gemcitabine therapy but show sensitivity to erlotinib.

Moffitt et al. later achieved successful molecular subtyping of both the epithelial cells and stroma of PDAC, leading to the identification of two distinct subtypes: normal and activated PDAC stroma. Notably, the activated subtype was associated with a poorer prognosis. The two subtypes specific to tumour were denoted as Classical and Basal-Like (67). The classical subtype is distinguished by the presence of overlapping genetic signatures, such as GATA6. The Basal-like subtype is correlated with a more unfavorable prognosis compared to the Classical subtype. However, it exhibits a more favorable response to adjuvant therapy.

In 2018, Puleo et al. examined the influence of the tumor microenvironment (TME) in PDAC. They categorised PDAC into five distinct clinical subtypes: Pure-basal-like, Stroma-activated, Desmoplastic, Pure-classical, and Immune-classical. Yet another classification distinguishes PDAC into Basal-like A/B, Classical A/B and Hybrids. Basal-like tumors are more aggressive; Basal-like A is associated with metastatic disease, and Basal-like B with resectable disease. Classical A/B tumors are frequently found in the early stage while Hybrids reveal the presence of multiple expression signatures (68).

Recent studies observed the coexistence of basal-like and classical subtype in PDAC. The intratumoral coexistence, which is increased during disease progression, inversely affects the prognosis and treatment based on subtypes. A comprehensive study of the dichotomous role of AP1 transcription factors (JUNB/AP1 versus cJUN/AP1) in PDAC subtype heterogeneity sheds light on the plasticity and stability of classical and basal-like neoplastic cells (69). It also highlights the importance of anti-tumor necrosis factor α (TNF-α) with gemcitabine chemotherapy which may provide a valuable strategy for a better treatment response in PDAC. The co-expression of tumor subtypes has been observed in approximately 90% of tumors using a multiplex immunofluorescence pipeline, based on the protein expression of PDAC subtype markers (70). The extensive intratumoral heterogeneity needs further characterisation in terms of intrinsic and extrinsic factors that dictate subtype heterogeneity. This will open up new prognosis and treatment options for PDAC patients (71).

## 2 TME complexity in PDAC

### 2.1 PDAC heterogeneity and plasticity

**Figure 3:**
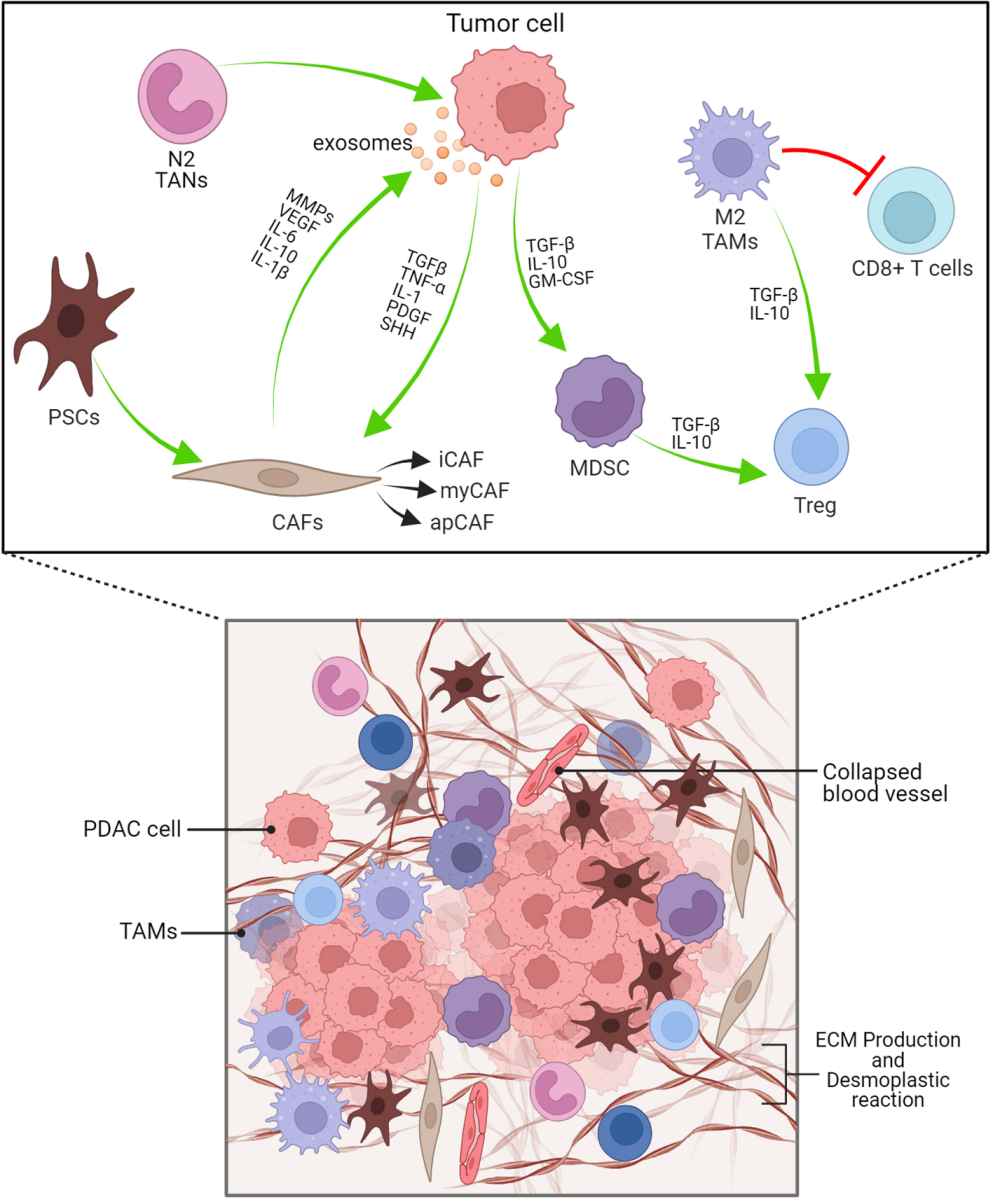
PDAC’s dense desmoplastic stroma and tumour microenvironment are depicted schematically here. The PDAC stroma is largely made up of CAFs, Macrophages, MDSCs and other immune cells. Exosomes produced from PDAC cells recruits and activates CAFs. Cytokines, TGF-β, IL-1, PDGF, SHH are cruicial for CAF activation. N2 TANs, myCAF, Tregs and Bregs have protumourigenic role. iCAF-inflammatory CAF, myCAF-myofibroblastic CAF, apCAF-antigen presenting CAF.

The cellular and humoural components make up the heterogeneous PDAC TME. In the cellular component, there are immune cells, endothelial cells, pancreatic stellate cells (PSCs), cancer-associated fibroblasts (CAFs) and myofibroblasts (**Figure 3**; **Table 1**). The humoural component is made up of collagen, fibronectin, and multiple soluble factors, including cytokines, chemokines, growth factors and complement components residing in the ECM (94–97). The interaction between these two components is essential for promoting tumour growth and the emergence of therapeutic intervention resistance. The development of an immunosuppressive TME, which allows the tumour to elude immune surveillance, is a feature frequently observed in PDAC.

**Table 1:** The immune cells in the tumor microenvironment of pancreatic ductal adenocarcinoma.

Phenotype	Cell type	Action	Effect	Reference
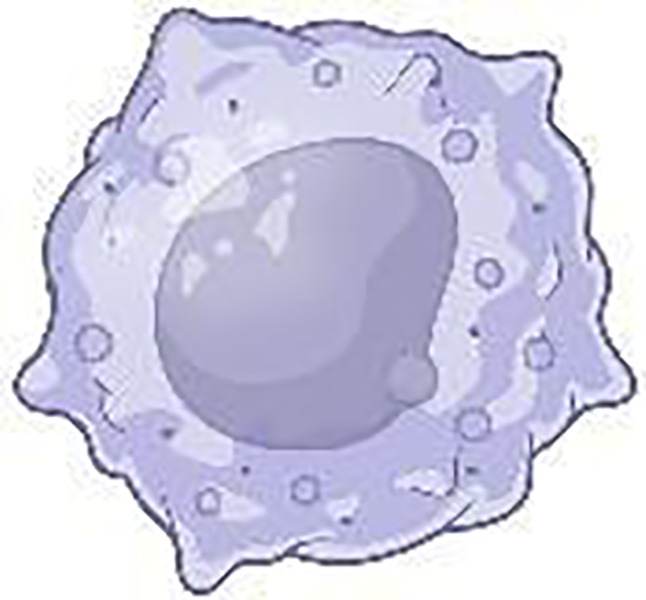	TAMs	Release various growth factors, cytokines; promote tumor cell invasion, induce angiogenesis, suppress antitumor immunity, and facilitate tumor cell metastasis Classified into two subtypes: M1 and M2	promote ADM and PanIN	(72) (73) (74) (75)
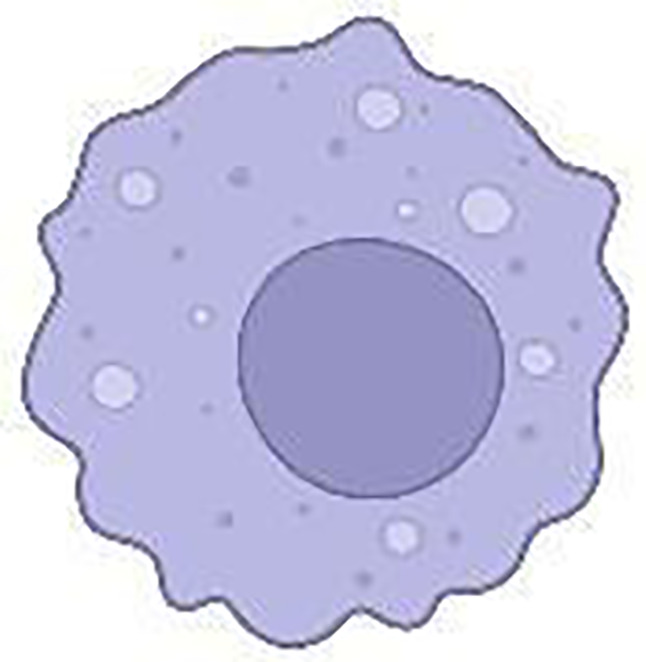	M1	Enhanced expression and release of IL-1β, TNF-α, IL-6, or IL-12	Antitumor and pro-inflammatory phenotype	(75)
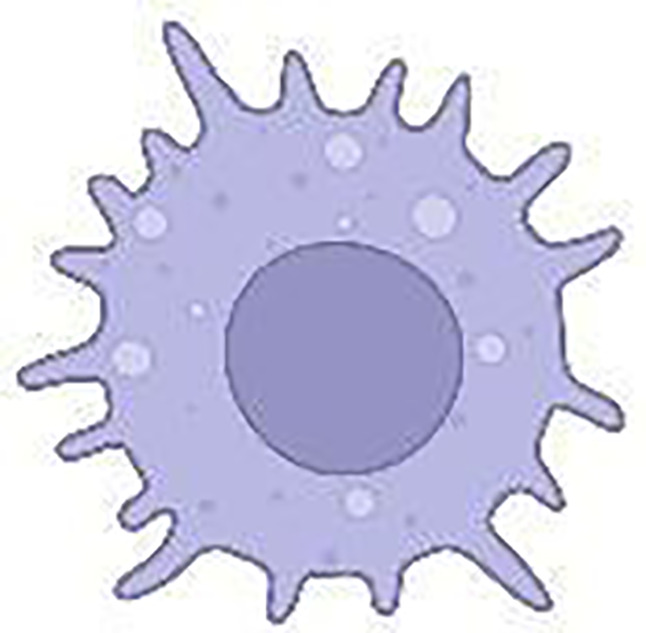	M2	IL-10, TGF-β, IL-6, PGE, CCL2, CCL17, CCL20	Protumor and anti-inflammatory properties, Inhibit CD8^+^ T cells activity, increases nodal lymphangiogenesis and poor prognosis	(75) (76) (77)
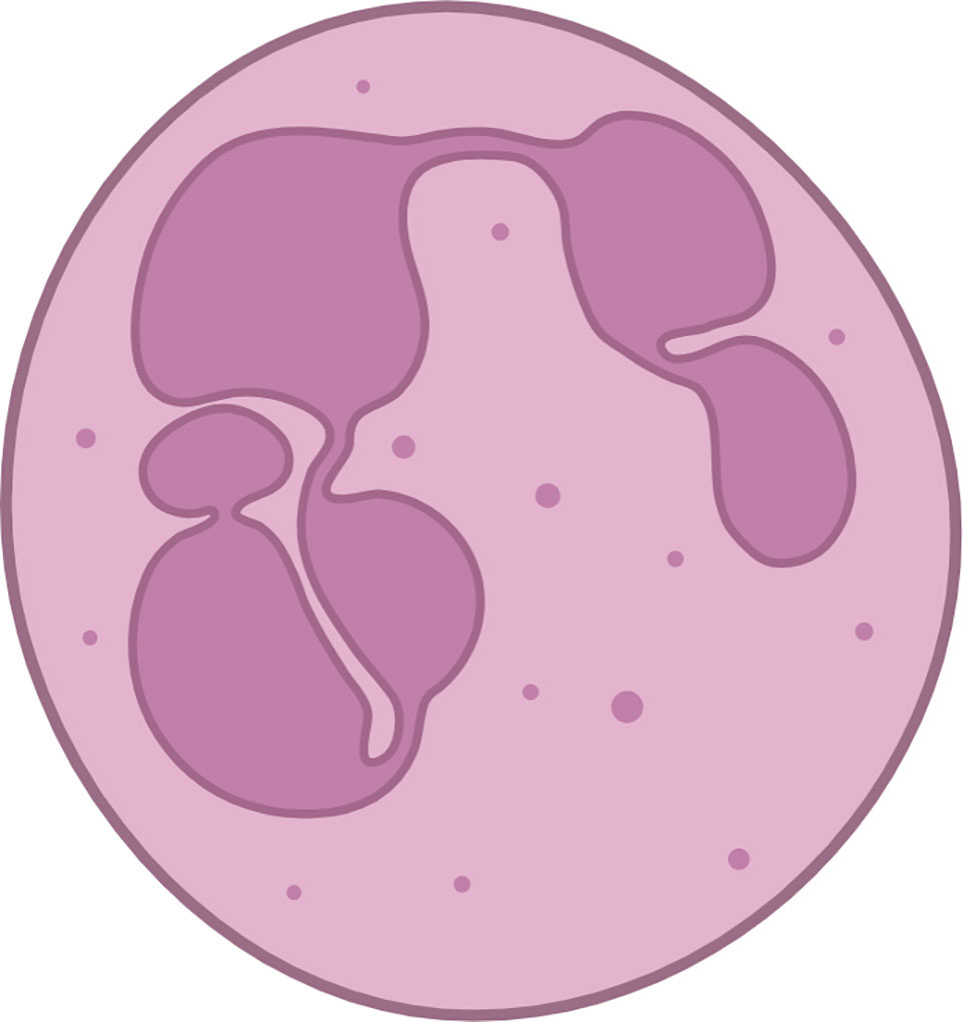	TANs	IFN-β, TGF‐β signalling	Differentiates into N1 or N2	(78)
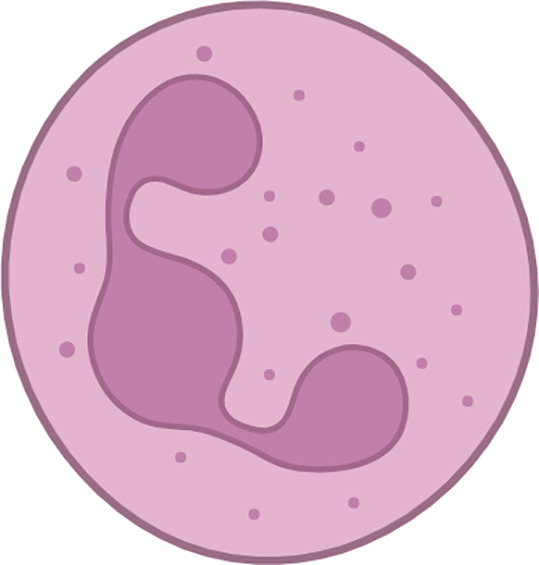	N1	IL-12, CXCL9, CXCL10, and CCL3	Recruitment and activation of CD8^+^ T cells, tumour suppressing	(79)
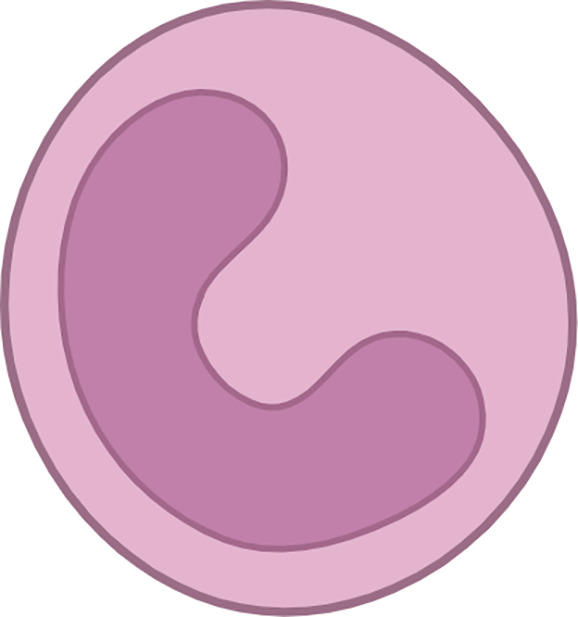	N2	VEGF, MMP-9	Tumour promoting by suppressing CTL	(80)
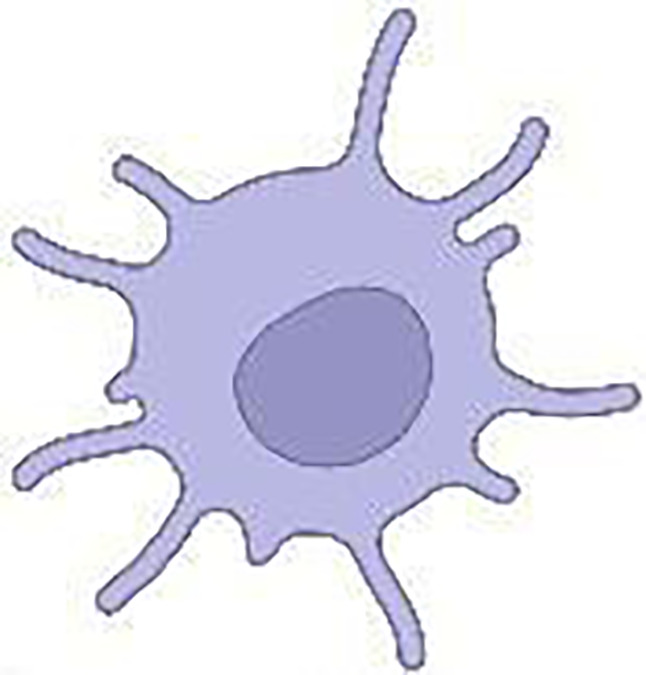	DC	Antigen presentation DCs infiltrating PDAC increases with TILs infiltration (CD4^+^ and CD8^+^)	Located in stroma and rarely in PDAC TME, improve overall survival	(81)
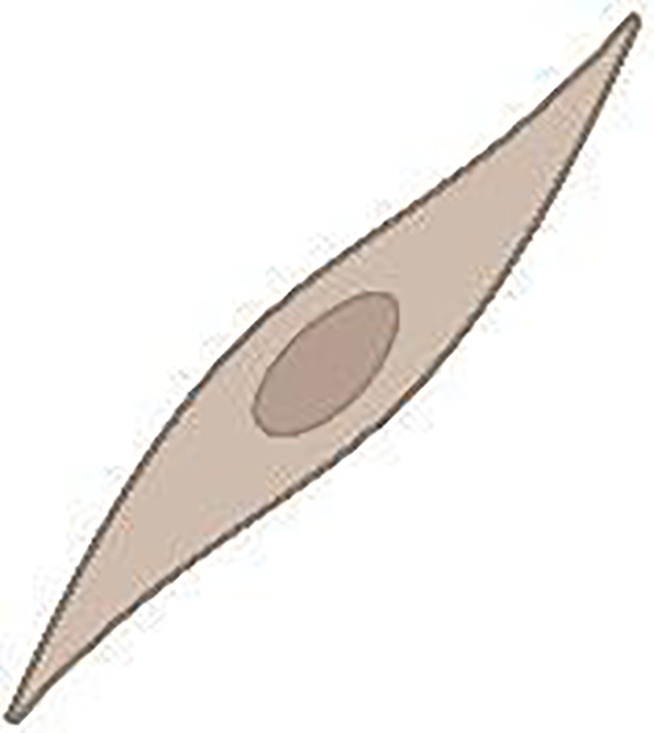	CAFs	IL-6, IL-11, TGF-β signaling ECM proteins include collagen, laminin, fibronectin IL-6, CXCL2, CXCL12, and CXCL8	Immune evasion by recruitment of Tregs Inhibitory TiME	(82) (83)
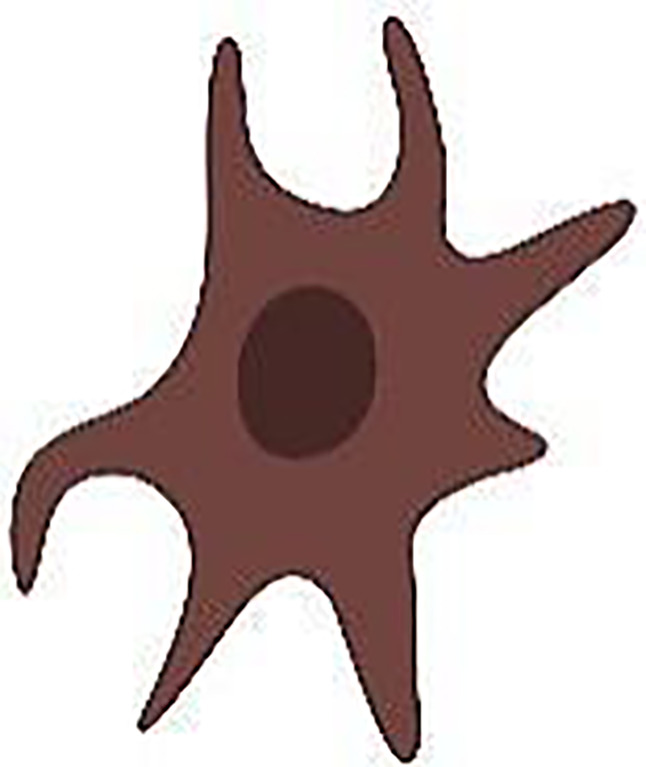	PSCs	Expressing alpha-smooth actin and produce growth factors, cytokines and ECM components	leads to desmoplastic reaction	(84)
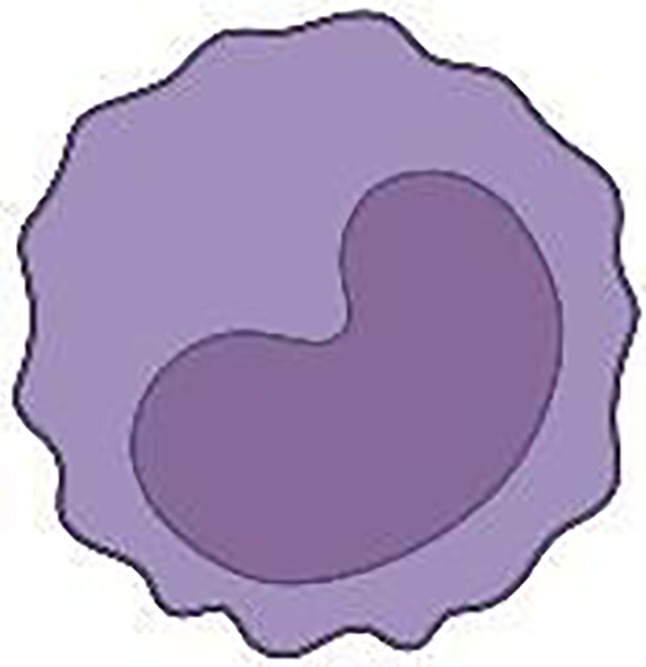	MDSC	Production of ROS, secretion of peroxynitrite and Arginase-1 Induction of Tregs Depletion of cysteine	inhibit the antitumor functions of T cells and NK cells	(85)
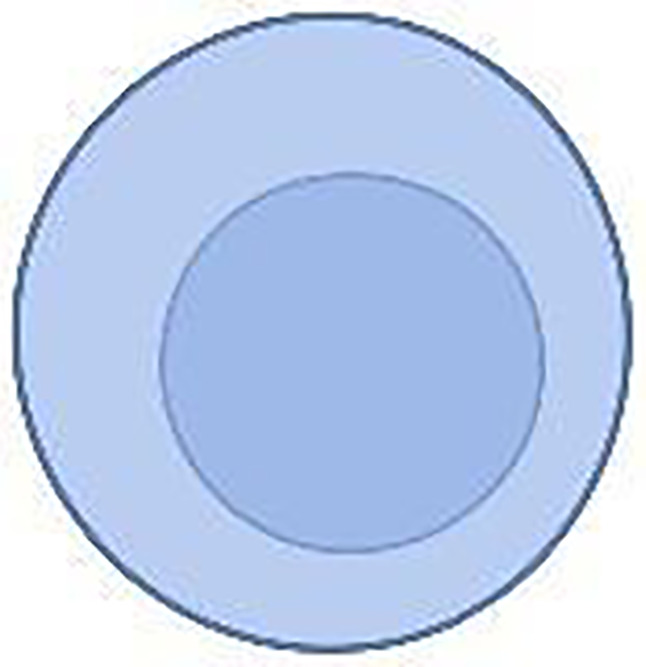	Tregs	Secrete immunosuppressive cytokines such as IL-10 and TGF-β FOXP3 protein expression and high levels of IL-2 receptor alpha chain CD25	immune evasion barrier for successful tumor immunotherapy	(86) (87)
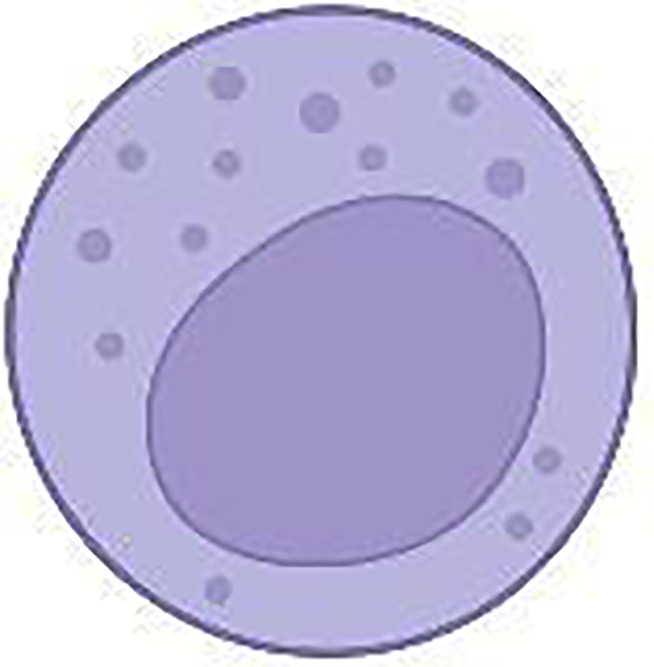	NK cells	Exhibit impaired killing of autologous PDAC cells due to NKG2D and DNAM-1 deficiency Increased percentage of NK cells in peripheral blood	Leads to recurrence-free survival	(88) (89)
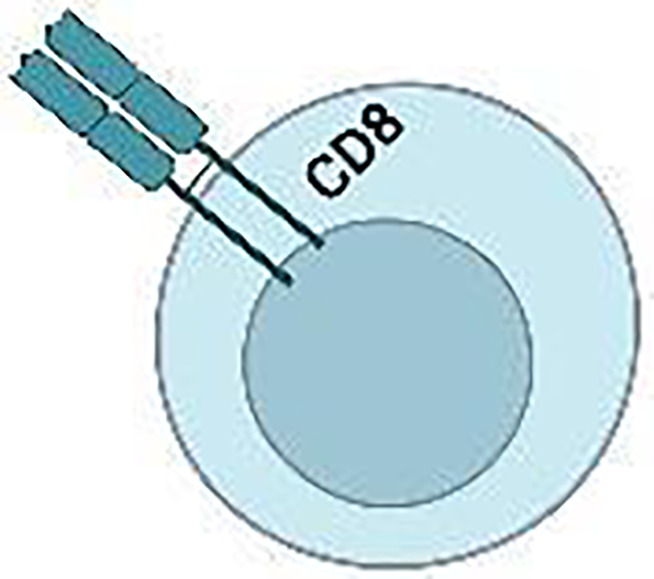	CD8+ T cells	IFN-γ, TNF-α, granzymes, FasL	Immunogenically hot tumor, which can respond better to immune checkpoint inhibitors	(81) (90) (91)
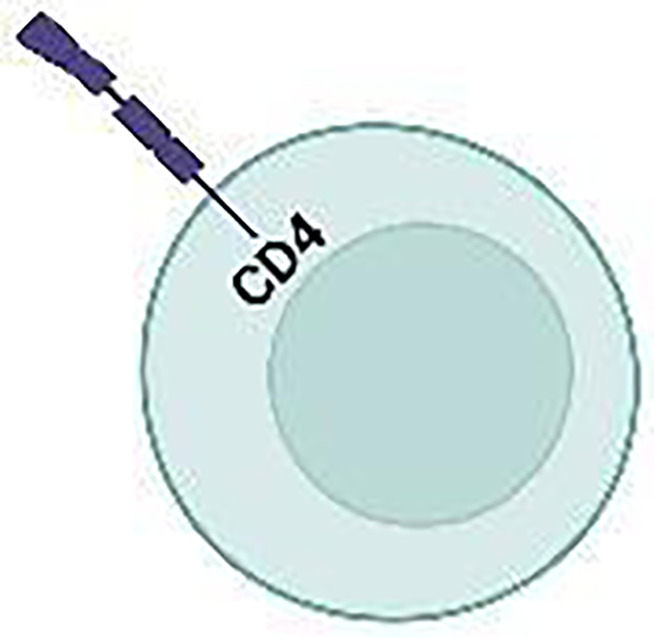	CD4+ T cells	IFN-γ, IL-2, increase CTL activity and IL-4, IL-5, IL-13, decrease CTL activity	Anti-tumor immunity and tumour tolerance	(91)
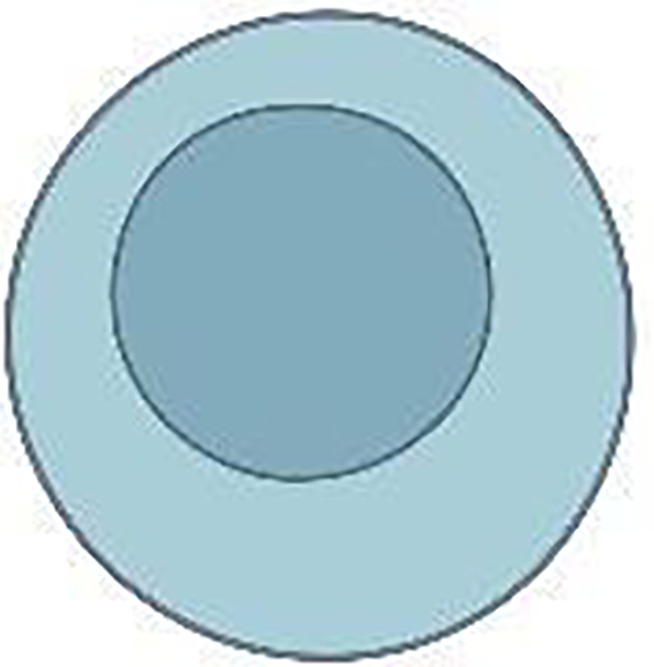	B cells	Infiltration of CD20^+^ B lymphocytes	Prognostic value diverged according to their spatial distribution in the tissue	(92)
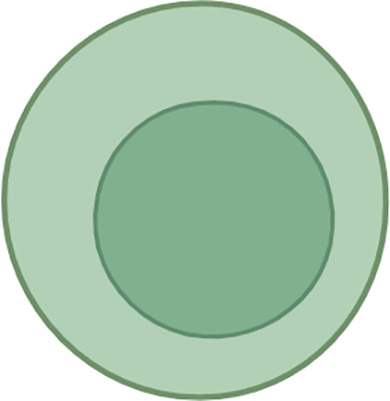	Bregs	IL-10, IL-35, IL-18	Enhance immunological tolerance	(93)

The human pancreas is comprised of exocrine (acinar), epithelial (ductal), and endocrine (α, β, δ, ϵ) cells. The plasticity of the pancreas is believed to be responsible for maintaining its homeostasis and promoting regeneration. Both acinar and ductal cells in the healthy pancreas can give rise to PDAC, though acinar cells appear to be more prone to oncogenic transformation (98). Acinar cells undergo a plastic trans-differentiation process known as acinar to ductal metaplasia (ADM), which can progress to PanINs, and eventually, adenocarcinoma (99), in response to specific macro- and microenvironmental stimuli, such as tissue damage, inflammatory factors, or stress conditions (98, 100), and become more vulnerable to activating mutations in the proto-oncogene KRAS. PanINs are the most frequent precursor lesions that are linked to the development of invasive PDAC among premalignant lesions with distinct histopathological features such as microscopic mucinous pancreatic ductal lesions with flat to papillary, micropapillary, or cribriform formation with severe nuclear atypia, loss of polarity, macronucleoli, and abnormal mitotic figures (86).

### 2.2 Inflammatory signatures

The etiology of PDAC would not be complete without highlighting the importance of inflammatory signals for initiation and progression of tumorigenesis. Inflammation and increased immune cell infiltration are common risk factors for human pancreatic cancer. Tumor-promoting inflammation, (101) is an integral part of neoplastic progression in PDAC. Chronic inflammation of the pancreas, known as pancreatitis, is a significant risk factor for the development of PDAC (102); the importance of environmental factors that cause chronic inflammation (e.g., smoking, heavy alcohol consumption, diet, and obesity) in pancreatic cancer is well established (103, 104). Inflammation promotes tumour formation, growth, progression, and metastasis (105).

The TME inflammatory cells and cancer cells are known to secrete several cytokines, such as IL-6, IL-10, IL-13, VEGF, and TGF-β (106). The anti-inflammatory TGF-β and IL-10, as well as the pro-inflammatory IL-1, IL-6, IL-17, and TNF-α, play a significant role in PDAC. Depending on the cross-talk between cancer cells and inflammatory cells, the ratio of pro- to anti-inflammatory cytokines in the TME constantly changes. Ling et al. demonstrated in a genetically engineered mouse model that oncogenic KRAS leads to a constitutive activation of NF-κB through IL-1α and p62 (107). Consequently, cancer cell-intrinsic inflammatory signalling networks generate a protumorigenic TME via the expression of cytokines that promote angiogenesis and the recruitment of immune and stromal cells. Several interleukins, including IL-6, were among those dysregulated by the depletion of NF-κB signalling. IL-6 class cytokines (e.g., IL-6, LIF, OSM, IL-11) are among the few regarded as master regulators of inflammation associated with cancer (108). Blocking the inflammatory cytokine IL-6 may improve the efficacy of anti-PD-L1 therapy by modulating immunological features of PDAC in murine models (109). It may enhance T cell trafficking and alter the tumor’s T cell population, as the ability of a patient to respond to checkpoint inhibitors is significantly impacted by T cell infiltration into tumours (110).

## 3 Immune suppressive microenvironment in PDAC

### 3.1 Myeloid cells

The immunosuppressive TME and cell types, a hallmark of pancreatic cancer, are thought to promote tumour invasion and growth. Myeloid cells develop from hematopoietic stem cells in the bone marrow through myelopoiesis. They are characterised by the expression of CD45 and CD11b surface markers. Subsequently, they undergo differentiation into discrete subpopulations, namely macrophages, granulocytes, mast cells, and dendritic cells, all of which are integral constituents of the innate immune system. Myeloid cell abundance in tumours correlates with worse clinical outcomes (111, 112).

The macrophages present within the tumour are commonly known as tumor-associated macrophages (TAMs). Two main phenotypes of macrophages, known as M1 and M2, are known for their ability to display plasticity. M2 polarised macrophages display immunosuppressive traits and a restricted adaptive immune response. Induction of an M1-like phenotype is typically seen to enable adaptive immunosurveillance. TAMs inhibit T-lymphocyte responses (113) and secrete cytokines that promote the tumor phenotype and metastasis (73, 114). In addition to their ability to directly induce T-lymphocyte apoptosis (115), TAMs produce arginase-1(72), a metalloenzyme that metabolizes and depletes the environment of arginine, an essential compound for T-lymphocyte proliferation (116, 117). Another major TAM subpopulation includes SPP1^+^ and C1QC^+^ TAMs, which on further characterization, showed enrichment for epithelial-to-mesenchymal transition (EMT) and a high angiogenesis score in SPP1^+^ TAMs, while C1QC^+^ TAMs were enriched for antigen presentation and phagocytosis (118). Granulocytes can be further categorised into three subtypes: eosinophils, basophils, and neutrophils. In the context of the TME, it is common for neutrophils and monocytes to exist in an immature state, which is commonly referred to as immature myeloid cells or myeloid-derived suppressor cells (MDSCs).

Myeloid cells and other immune cells infiltrate the PDAC TME, resulting in a state of local inflammation (119) in which tumour cells interact with infiltrating immune cells. However, for a transformed cell to survive, it must attain an immunosuppressive phenotype, such as downregulation of MHC class I expression and upregulation of programmed cell death receptor ligand-1 (PD-L1) and CD47, which hinder the anti-tumor immune response by engaging and suppressing the activated T cells and relaying ‘don’t eat me’ signal to the phagocytic macrophages, respectively (120, 121). Constitutively active KRasG12D regulates autophagy-induced MHC class I downregulation, which is a major mechanism that PDAC cells employ to escape immune surveillance (122, 123). **Table 1** provides the immune cell composition and its effect in the PDAC TME.

### 3.2 Cancer-associated fibroblasts

In addition to myeloid immune cells, fibroblasts in stromal components, known as Cancer-associated fibroblasts (CAFs), are an important TME component, including myofibroblastic CAFs (myCAFs), immunogenic CAFs (iCAFs), and antigen-presenting CAFs (apCAFs). PDAC has a unique fibrotic TME with desmoplastic stroma, abundant in ECM proteins produced by CAFs that represent a significant proportion of the cellular composition in the PDAC stroma, ranging from 15% to 85% of stromal cells (124) (**Figure 3**). CAFs, immune cells, cytokines and chemokines accumulate in the TME of primary and metastatic PDAC, exacerbating the development of an immunosuppressive phenotype (86, 125). CAFs create a physical and metabolic barrier via ECM proteins, thereby diminishing the effectiveness of therapeutic interventions against PDAC by increasing the interstitial tumor pressure that impairs vascular function (126–128). Additionally, CAFs facilitate tumour growth and invasion (129–132) and contribute to chemotherapy resistance by the presence of hyaluronan (18, 33, 127). The role of CAFs in immunosuppression (133), tumour metabolism (134), and secretion of inflammatory factors such as IL-1β, potential initiator of NF-κB signalling (135), have been studied. Therefore, the elimination of CAFs from the TME has the potential to serve as a possible therapeutic approach for the treatment of PDAC (131, 136).

### 3.3 Extracellular matrix

Increasing desmoplasia, which frequently matches or exceeds the tumor’s epithelial component, is a hallmark of PDAC progression. The ECM provides physiological signals to neighboring cells in all tissues. The accumulation of ECM proteins is prevalent in solid tumours including PDAC and is referred to as a desmoplastic reaction (137). TME can modulate interstitial fluid pressure (33, 126) and reduce the density of blood vessels within tumours (33). Collagens, integrins, proteoglycans, glycoproteins, and proteases dominate the ECM of PDAC. These components interact with cancer cells through a variety of mechanisms (138, 139). Collagens Type I, III and IV are the most prevalent of these constituents. Collagens are active components in PDAC stroma with not for just structural support, but have a direct effect on the growth, survival, and spread of cancer cells (140); patients with higher level of fibrillar collagen have lower overall survival rate (141). α_v_β_6_, an epithelial integrin, is upregulated in PDAC (142). Galectin-1 (GAL1), along with other glycoproteins such as periostin and fibulin, has been found to be upregulated in the PDAC TME and is poorly expressed in long-term (10 years) PDAC survivors (143).

CAFs, which originate primarily from PSCs and bone-marrow derived mesenchymal stem cells, are a major regulator of the ECM (144). PSCs are primarily located in the vicinity of pancreatic glands and possess the capability to produce ECM proteins, matrix metalloproteinases (MMPs), and MMP inhibitors, which play a crucial role in regulating ECM turnover (145). PSCs can be activated by pro-inflammatory cytokines, oxidative stress, hypoxia, hyperglycemia, and heightened interstitial pressure (146). Activated PSCs can secrete growth factors such as TGF-β1, PDGF and VEGF (147) to promote pancreatic cancer cell growth, decrease apoptosis, and increase invasion (148). PSCs are the primary source of collagen in tumour stroma, secreting ECM proteins such as α-smooth muscle actin and collagen. Reducing myofibroblasts and ECM in PDAC in vivo can inhibit tumour growth and improve chemotherapy sensitivity.

## 4 Interplay between innate and adaptive immune mechanisms in PDAC

PDAC is immunologically heterogeneous; this heterogeneity exists between cells within PDAC. CD8^+^ cytotoxic T lymphocytes (CTL) and CD4^+^ T cells are the effector tumor infiltrating lymphocytes (TILs) observed in resected cancer tissue and are believed to participate in the host immune response against cancer which is considered a positive prognostic marker (149). Out of the total T lymphocytes (CD3), >80% are CD8^+^ T and CD4^+^ T Cells (150). The immune cells that target tumors are CTLs. CTLs use the Fas-FasL and perforin–granzyme pathways as major effector mechanisms of cytotoxicity; loss of Fas expression in PDAC tumours result in cancer immune evasion (151). PSCs produce elevated amounts of ECM, driving a fibrotic tissue that entraps infiltrated T cells, alongside immunosuppressive cytokines and expression of PDL-1. Pancreatic cancer cells avoid T cell killing by downregulating Fas, exhibiting low tumour mutational burden, expressing PDL-1 and secreting growth factors and cytokines that recruit immunosuppressive cells. CTLs are localised along the invasive margin of the tumour border or trapped in the surrounding fibrotic tissue but are not present within the tumour core. Moreover, infiltrated CD8^+^ T cells in PDAC tumours often display minimal signs of activation (152). MDSCs express PDL-1 and suppress T cells functions by several mechanisms, including depletion of arginase 1, the release of reactive oxygen species, and secretion of cytokines. Tregs directly suppress T cells, express cytotoxic T-lymphocyte-associated protein 4 (CTLA-4) and secrete cytokines such as TGF-β and IL-10. TAMs play a role in sequestering T cells at the periphery and secrete immunosuppressive cytokines (91). Tumour-derived cytokines and chemokines drive recruitment of myeloid cells to the TME. These cells, which include TAMs and MDSCs, block the recruitment and priming of T cells, resulting in T cell exclusion within the TME (153).

There is considerable infiltration of CD20^+^ B lymphocytes in the TME of human PDAC, unlike normal pancreatic tissue (92). There is a distinct spatial heterogeneity for B cells either in ectopic lymph nodes like tertiary lymphoid structures, or interspersed at the tumour–stroma interface. In addition, B cells produce anti-tumor antibodies and present tumor antigens to T cells to improve the cancer immunosurveillance. B cells in the TME respond to tumor-associated antigens by secreting IgG1 antibodies to activate the complement system, and phagocytosis by NK cells and macrophages (154). Alternatively, regulatory B cells (Bregs), dispersed inside the TME, contribute to the dampening of anti-tumor immune responses by secreting anti-inflammatory cytokines (IL-10 and IL-35), which promote tumor growth and metastasis (93). It appears that innate immune cells such as macrophages and neutrophils have a larger role to play in PDAC than the adaptive immune mechanisms.

### 4.1 Regulatory T cells

In PDAC, regulatory T cells (Tregs) play a major role in tumour immune suppression. Through immunohistochemistry, they can be identified based on forkhead box protein 3 (FOXP3) expression and high levels of IL-2 receptor α chain CD25 in tumour tissues. There is sufficient evidence that Tregs are the primary barrier to an effective tumour immunotherapy (87). In fact, Tregs are significantly increased in the blood of PDAC patients as well as in the pancreatic tissue (155). They are recruited to tumour sites, where they inhibit antitumour cytotoxic response by binding to DCs and preventing DCs from activating CD8^+^T cells (156). From the premalignant to the invasive stages of PDAC, Tregs aid in suppressing the immune response against PDAC cells (157). In addition, it appears that a high Treg prevalence in PDAC is linked to a poor prognosis and weak PDAC differentiation (158). Single cell RNA seq studies revealed that activated TME is defined by the presence of Tregs, FGF, TAMs (SPP1^+^, GRN^+^), M2 like macrophages; in contrast, patients with normal stroma show M1-like macrophages, increased effector and exhausted T-cells (159).

Using the KC mouse model, a model where KRAS genetic changes are brought in for the development of pancreatic cancer, the immune cell infiltration at different stages of PDAC, including normal pancreas, PanINs, and invasive carcinoma, were examined (160, 161). Tregs and MDSCs predominated the immune infiltrate in the early PanIN stages. When the disease reached the PDAC stage, CD4^+^ and CD8^+^ cells were infrequently found and the existing CD8^+^ cells were not activated, suggesting an immunosuppressed TME (160). Strong inverse correlations between MDSCs and CD8^+^ T-lymphocytes at all disease stages imply that MDSCs are a key player in tumour immunosuppression (160).

Clinically, pancreatic cancer frequently contains T lymphocytes which surround the pancreatic lesion; CD8^+^ cells are elevated in the circulation of PDAC patients (162). PDAC has a high percentage of CD4^+^ Tregs, which support an immunosuppressive phenotype. They are typically found in the stromal regions of the tumour and rarely in conjunction with tumour epithelial cells (157). Treg accumulation is correlated with the progression of both the major preneoplastic lesions, PanINs and IPMN, in clinical samples of pre-malignant lesions (157). In murine models of PDAC, an association between Treg infiltration and the growth of pancreatic cancer is established. When syngeneic C57BL/6 mice are subcutaneously injected with mouse pancreatic tumour cells from Pan02, the spleen and tumour-draining lymph nodes of these mice exhibit a marked increase in Tregs (163). The CCR5 receptor, which is preferentially expressed by Tregs, is ligated by tumour cells in murine as well as human PDAC (164). Growth of PDAC is inhibited by CCR5 mediated blockade of Treg accumulation.

### 4.2 Regulatory B cells

Tumor-infiltrating B lymphocytes in PDAC differentiate into Regulatory B cells (Bregs) that produce IL-10 or IL-35 with the help of other immune cells such as Tregs and MDSCs, cytokines IL-18, CAFs, tumor-associated antigens, damage-associated molecular patterns, hypoxia, pancreatic microbiota, and metabolites in the TME (165, 166). A high number of IL-10/IL-35-producing Bregs are observed in the PDAC stroma of KPC and KC murine models and PDAC patient samples (93). IL-18 promotes Breg differentiation and enhances immunological tolerance, leading to the development and metastasis of PDAC (46). In addition to IL-18, other chemokines such as CXCL13 and CCL21, are responsible for B-cell migration and accumulation within tumors (93).

## 5 How tumour cells shape innate immune response in PDAC progression

### 5.1 Tumour intrinsic chemokines and cytokines

Cancer stem cells (CSCs) in PDAC have the ability to self-renew, differentiate into numerous lineages, initiate tumourigenesis, and resist conventional cancer therapy. CSCs are characterized by specific cell surface markers, CD44^+^CD24^+^ESA^+^ (167). Pro-inflammatory cytokines are involved in the CSC self-renewal process (168). Following the development of pancreatitis, the number of CSCs in the circulation greatly increased. However, treatment with the anti-inflammatory drug, dexamethasone, lowers the level of CSCs in the circulation. Thus, inflammation plays an important role in the spread of pancreatic CSCs and perhaps even in PDAC metastasis.

Tumour-derived cytokines and chemokines in PDAC set up the immunosuppressive cellular network by attracting myeloid cells to the TME. TAMs and MDSCs contribute to T cell exclusion from the TME by inhibiting their recruitment and priming. By secreting cytokines, chemokines, and other factors such as GM-CSF, CSF-1, IL-3, CXCL12, and CCL2, TAMs and MDSCs can shape the TME in ways that promote or inhibit tumour growth and survival.

Acinar cell trans-differentiation into duct-like cells, known as acinar to ductal metaplasia (ADM), is the first histologically distinct event during PDAC pathogenesis (98, 99, 169). ADM is required for pancreatic regeneration by the acinar cells and is accompanied by a loss of polarity or contact between cells or with the ECM. However, pro-inflammatory cytokines prevent the acinar reversibility in the presence of oncogenic Kras and advance ADM to lesions PanIN (99). TNF-α and RANTES (Regulated on Activation Normal T Cell Expressed and Secreted) are two pro-inflammatory cytokines secreted by TAMs that cause ADM by triggering NF- κB signalling and the expression of MMPs (170, 171).

The functional relevance of the chemokines in PDAC and their association with the NF-κB pathway has been studied (172). TAMs also secrete IL-6 and promote STAT signaling resulting in tumour growth and progression (173, 174). The initial secretion of cytokines such as PDGF and TGF-β, recruits additional lymphoid and myeloid subsets into the TME, which then secrete more TGF-ß1, CTGF, high mobility group box protein 1 (HMGB1), IL-10, IL-1α, IL-1β, IL-8, TNF-α, and CCL18 depending upon their activation status (175), resulting in chronically inflamed tissues. Signaling through a family of G-protein coupled receptors is an additional important stimulus for the infiltration of these immune cells into the PDAC tissue (176).

Numerous chemokines are described in relation to PDAC pathogenesis and therapy resistance. PDAC cells produce chemokine CCL2 or monocyte chemotactic protein 1 (MCP1), a proinflammatory chemokine that binds to CCR2 and CCR4 under normal conditions (177). CCL2 is found to be highly expressed in Basal like subtype compared to Classical subtype and recruits TAMs to the TME (178). This basal expression is further increased when the cells are stimulated with IL-1, TNF-α or FAS ligand (179). Furthermore, the regulation of CCL2 expression in PDAC cells are attributed to the NF-κB pathway (177, 179, 180). CXCL8, or IL-8, is a chemokine produced by many cell types. IL-8 also binds to CXCR1 and CXCR2, with a higher affinity for CXCR1. In addition to angiogenic functions, IL-8 mediates phagocytosis and chemotaxis.

## 6 PDAC aggressiveness and immune suppression

### 6.1 Innate immune-driven PDAC aggressiveness

Immune cell fractionation in PDAC revealed a higher proportion of innate immune cells than adaptive immune cells (8). PDAC tissue contains an abundance of macrophages, MDSCs, DCs, and neutrophils. Single-cell RNA sequencing (RNA-seq) data revealed that macrophages are the predominant immune cells among the CD45^+^ population in PDAC (8). Neutrophils also contribute to significant portion of the immune cell infiltrate observed in PDAC (181). Neutrophils are transformed into tumour-associated neutrophils (TANs) after migrating into tumour tissues. TANs were identified as Ly6G^+^CD11b^+^ cells (182), and further classified as N1 (tumour suppressing) or N2 (tumour promoting) phenotype (100) and are associated with poor prognoses in PDAC (183). Neutrophils are recruited to the PDAC TME via multiple tumour-secreted chemokines including CXCL1, CXCL2, CXCL5, and CXCL8. They respond to these chemokines by the expression of the CXCR1 and CXCR2 CXC receptors. Tumour size in PDAC correlates with the level of CXCR2 expression (184). Myeloperoxidase^+^ (MPO^+^) neutrophils and CD11b^+^Ly6G^+^ MDSCs infiltration into tumours is reduced in CXCR2 knockout PKF [mice with conditional Kras^G12D^ mutation and knockout of TGF-β receptor type II (Tgfbr2), (LSL-KrasG12D/+; Tgfbr2flox/flox, Ptf1a-Cre] mice compared to control animals (185). CXCL1, CXCL2, and CXCL5 secretion from tumour cells is elevated in the KPC (LSL-KrasG12D/+; LSL-Trp53R172H/+; Pdx1-Cre) mouse model, in comparison to the normal pancreas (186). Another study demonstrated that CXCL5 has the greatest increase in human PDAC and correlated with both tumour-infiltrating CD15^+^ granulocytes and neutrophil elastase^+^ (NE^+^) granulocytes (187). Neutrophil depletion has been shown in multiple PDAC studies to reduce tumour growth and metastasis. Importantly, in wound healing and transwell assays *in vitro*, neutrophils derived from PDAC patients significantly promoted the migration and invasion of pancreatic cancer cells, whereas neutrophils derived from healthy individuals did not (188). In addition, Neutrophil to Lymphocyte Ratio correlates with a poor prognosis in patients with resectable and unresectable pancreatic cancer (187, 189–191). PDAC patient outcomes also correlate with the presence of neutrophils within the tumour. Neutrophil marker CD177 is inversely associated with overall survival in patients with PDAC (192). Patients with PDAC who have tumour-infiltrating neutrophils with high levels of CD66b^+^ have significantly lower survival rates (181). In human PDAC tissues, TAN-derived TGF-β induces EMT in human lung cancer tissues through the TGF-β/Smad pathway, contributing to carcinogenesis (193, 194). Another study indicates that inhibition of CXCR2 decreases TAN accumulation, and inhibits PDAC metastasis in mice (186, 195).

DCs are uncommon in the TME of pancreatic cancers and are located at the tumour’s periphery (196). Systemically, PDAC patients have decreased levels of blood DCs (197). Notably, higher levels of circulating DCs are associated with improved survival in PDAC patients (197, 198). In addition, surgical removal of the pancreatic tumour improved blood DC function, supporting a tumour-derived effect on immune function of DCs (199). During disease progression, the immune response of the host to pancreatic cancer is reported to shift from immune surveillance to immune tolerance. CXCL17 and intercellular adhesion molecule 2 (ICAM2) appear to mediate this polarisation (200). In addition, tumour-derived cytokines such as TGF-β, IL-10 and IL-6 have been shown to inhibit DC survival and proliferation (201). The proliferation of immature myeloid cells in the bloodstream and spleen may further compromise the immune response. The level of circulating MDSCs is increased in PDAC, which may promote tumour progression (202, 203). MDSCs inhibit DC activation in pancreatic cancer by producing nitric oxide (NO) (204).

Innate lymphoid cells (ILCs) are innate immune cells that bridge between innate and adaptive immune system. Group 2 ILCs (ILC2s) are activated by IL-33, which have differential roles in PDAC development and progression; ILC2 activation recruits T cells to boost anti-cancer immunity in PDAC tissues via recruitment of CD103^+^ DCs (205). However, yet another study demonstrated that IL-33-treated ILC2s produced IL-10 and played a protective role in islet allograft survival (206). These results indicate that ILC2s are a highly dynamic cell type and their phenotypes and functions are controlled by the TME. In the TME, immunosuppression is observed where hypoxia converts ILC2s to IL-10^+^ ILCregs, helping to form a tolerogenic state in pancreatic cancer (9). ILCs recruit CD8^+^ T and memory T cells in PDAC; ILCs are also able to help CD108^+^ B cells migrate to tumour locations (207).

### 6.2 Innate-immune driven immune suppression in PDAC

PDAC is notoriously resistant to immunotherapy, such as cytokine therapy, adoptive T cell therapy, and checkpoint blockade strategies (208–210) Failure of these therapies has been attributed to a lack of CD8^+^ T cells and severe immunosuppression in the TME of PDAC (45, 211, 212). The presence of excessive fibrosis in the TME hinders the infiltration of adaptive immune cells (127).

At the early PanIN stages, Tregs and MDSCs dominate the immune infiltrate. As the disease progresses, CD4^+^ and CD8^+^ cells are inconsistently found; existing CD8^+^ cells display a lack of activation, suggesting an immune suppressed TME (160). At all stages of disease, there is a strong inverse correlation between MDSCs and CD8^+^ T-lymphocytes, suggesting that MDSCs are a mediator of tumour immunosuppression (160).

Conventional DCs (cDCs) have been identified as important mediators of antigen priming and T cell activity, with Batf3/Irf8-dependent CD103^+^ CD24^+^ cDC1s responsible for CD8^+^ CTL cross-priming. Moreover, Irf4-dependent CD11b^+^ CD172a^+^ cDC2s are implicated in the priming of CD4^+^ T helper cells (Th) (213). cDCs have also been implicated in T cell-dependent tumour killing and immunotherapy response (214–218). Nonetheless, it has been reported that the levels of circulating MDSCs are elevated in pancreatic cancer, which may promote tumour progression (202, 203); MDSCs produce NO, which inhibits DC activation (204). Depending on microenvironmental stimuli, DC can differentiate into distinct subpopulations, leading to proliferation of myeloid DCs that induce Th1 cell activation, or plasmacytoid DCs that facilitate immunosuppressive T cell development. Tumour-derived cytokines have been reported to induce a tolerogenic plasmacytoid DC phenotype (201). Furthermore, recent data suggest the existence of a specific subset CD11b^+^ DCs that foster an immunosuppressive TME, which favors metastatic progression through the expansion of Tregs and suppression of CD8^+^ T cells (219). These findings indicate that PDAC is characterized not only by a reduced number of DCs, but also by complex modulation of DC subpopulations, which affects tumour development.

### 6.3 Complement system

The complement system is a crucial mechanism that connects innate immunity to adaptive immunity and aids the body in combating foreign pathogens and abnormal host cells (220). The complement system can be activated by three distinct pathways: classical, alternative, and lectin. The three pathways converge on the cleavage of complement component C3 into subunits C3a and C3b (C3 convertase) and C5 into fragments C5a and C5b (C5 convertase). As inflammatory mediators or anaphylatoxins, C3a and C5a, cause inflammation by causing histamine release and by activating immune cells such as neutrophils, eosinophils, and macrophages (221). Malignant tumours have increased complement protein expression (222). Activation of the complement system in the TME promotes tumourigenesis (222). PDAC tissue shows an upregulation of C3 and C5, producing more anaphylatoxins (223, 224), C3a and C5a, which upregulate inflammatory mediators and cytokines and cause direct stimulation of TNF-α and IL-1 (220). In addition, these anaphylatoxins increase the recruitment of macrophages in the TME (225).

The complement system has an important role to play in PDAC. The expression of complement regulatory proteins/receptors, CD46, CD55, and CD59, is well established in PDAC cell lines (226). Properdin, the only known up-regulator of the alternative pathway, is highly expressed in the early stages of PDAC; its decreased expression in samples from patients with late-stage PDAC has been reported (227). Neutrophils are known to secrete properdin, which is stored in their granules (228). Elevated properdin expression in PDAC patients with increased neutrophil infiltration is more likely to associate with classical subtype and higher overall and disease-free survival. Properdin induces apoptosis in basal-like pancreatic cancer cell lines, suggesting its anti-tumourigenic role in PDAC (229). Studies have also reported that properdin can recognize cancer cells and play a protective role during tumourigenesis (230). Alternatively, properdin level is strongly down-regulated in PDAC serum (231). The inhibition of complement activation promotes cancer cell immune evasion and seems to hamper the efficacy of cancer immunotherapy.

## 7 Targeting innate immunity in PDAC

Existing therapies for patients with PDAC include surgical resection, chemoradiation therapy, and immunotherapy; however, only a small percentage of patients benefit from these treatments. Single-cell RNA-seq studies on the PDAC TME show innate immune cell dominance, which can be directly activated by many cytokines without antigen presentation, unlike adaptive immunity. Given the predominant infiltration, decreased antigenicity, and instant activation, innate immunity may be more important than adaptive immunity in the PDAC immune TME.

On the basis of a growing comprehension of the role of TME in PDAC, neutrophils have emerged as a possible therapeutic target. Targeting neutrophils in PDAC has shown encouraging results in a number of preclinical studies that utilised CXCR2 inhibitors or Ly6G antibodies (187). The preference for CXCR2 as a target may possibly arise from the fact that blocking CXCR2 affects not just the CXCL5/CXCR2 axis but also additional CXCR2 ligands, such as CXCL1-3 and CXCL6-8.

TAMs are one of the most important regulators in the PDAC TME. Depletion of TAMs could dramatically decrease tumourigenesis (232); inhibiting M2 macrophage polarisation is essential for preventing PDAC development, enhancing antitumour immunity, and even clinical treatment (233). New developments in macrophage adjustment have been put forth, such as blocking CSF-1/CSF-1R, CD40 agonists, and other agents, which are helpful in re-educating TAMs from their M2 state to M1. It is currently possible to effectively halt tumour growth and cure tumours owing to an expanding variety of macrophage-targeting strategies. When combined with standard therapy and immunotherapeutic drugs, the blocking of CSF-1/CSF-1R activation can be a potential strategy for treating PDAC by decreasing the TAM population (234). Reprogramming the M2 phenotype of TAMs can significantly change the immunological status of the TME and reactivate the immune system’s antitumour activity.

The phase II clinical testing of multiple antibodies against CSF1/1R in PDAC patients has been undertaken (235). The efficacy of cabiralizumab (Five Prime), a humanized IgG4 mAb against CSF1R, together with the anti-PD1 antibody, nivolumab, in patients with advanced/metastatic PDAC who progressed after first-line chemotherapy (NCT03336216) was evaluated (236). Similarly, another phase Ib/II trial evaluated a fully humanised IgG2 monoclonal anti–CSF1R antibody, AMG 820 (Amgen), in combination with pembrolizumab, on patients with metastatic PDAC (NCT02713529). Both trials failed to reach their effectiveness goals despite exhibiting target-specific alterations, such as the decrease in monocytes. The failure may have been related to the normal stroma association of the CSF1/CSF1R signaling and non-specific targeting based on the expression of CSF1R seen across all myeloid cells. In another phase Ib/II trial that included patients with metastatic PDAC, limited activity was observed with the anti–CSF1 antibody, lacnotuzumab (Novartis), given in combination with anti-PD1 spartalizumab (NCT02807844) (237).

HA is highly overexpressed by tumour cells and CAFs in PDAC; enzymatic depletion of HA using PEGylated hyaluronidase improves therapeutic effectiveness (37). Based on the expression levels of HA, clinical phase I/II study (NCT01839487) revealed robust response rates for patients. However, when combined together, nab-paclitaxel and gemcitabine failed to prolong progression free survival (238).

High levels of integrin molecule CD11b/CD18 on myeloid, cell surface, which is essential for their trafficking and cellular activities within inflammatory tissues, make them amenable to therapeutic targetting. ADH-503 is a small-molecule agonist that partially activates CD11b, causing TAMs to repolarize, fewer immunosuppressive myeloid cells to infiltrate the tumour, and improve DC responses. As a result, checkpoint inhibitors are now effective in PDAC models that were previously resistant to their effects and antitumour T cell immunity is improved. These results show that molecular inhibition of CD11b alters immunosuppressive myeloid cell responses and may overcome the limitations of existing clinical approaches to immunotherapy resistance (239).

DC vaccination has emerged as a novel strategy to prime host anti-tumour immunity (240). Specifically, the combination of a DC vaccine with gemcitabine led to eradication of orthotopic tumours and provided durable protection against PDAC in mouse models (241).

ECM plays a significant role in PDAC tumour growth, metastasis, and resistance to therapy. Accumulating preclinical studies with patient-derived specimens suggest that targeting the dense desmoplastic ECM proteins of PDAC may offer the potential for clinically useful treatments. In clinical practice, it has not yet been possible to successfully target the ECM to improve overall survival.

## 8 Recent clinical trials

Antibodies against immune checkpoints, such as anti-PD-1/PD-L1 and anti-CTLA-4, brought transformation in the treatment of several malignancies, but failed to elicit effective anti-tumour response in PDAC patients (19). A phase II clinical trial (NCT02879318) assessed the safety and efficacy of combination chemotherapy (Gemcitabine and nab-paclitaxel) with immune checkpoint inhibitors (durvalumab; PD-L1 inhibitor) and tremelimumab (CTLA-4 inhibitor), which did not improve survival rate significantly (242). Modified FOLFIRINOX (Folinic acid, fluorouracil, irinotecan, and oxaliplatin) with Sintilimab (human IgG4 monoclonal antibody for PD-1) used in a clinical trial (NCT03977272) did not show any survival benefit (243). Another phase II trial (NCT032124250) evaluated the efficacy of nivolumab (anti-PD-1) and/or sotigalimab (CD40 agonistic antibody) with gemcitabine/nab-paclitaxel (chemotherapy) in patients with first-line metastatic PDAC (244). The overall survival rate was 57.7% in nivolumab/chemotherapy group compared to 48.1% observed in sotigalimab/chemotherapy and 41.3% in nivolumab/sotigalimab/chemotherapy treatment regimen. Granulocyte-macrophage colony-stimulating factor (GM-CSF)-secreting allogeneic pancreatic tumour cell (GVAX) immunotherapy and ipilimumab did not improve overall survival, but clear biologic effects on peripheral and intratumoural immune cells were observed, such as increase in T cell activation markers, peripheral T helper and cytotoxic effector memory cells, and decrease in naïve cytotoxic T cells and increase in M1 macrophage content (245). A Phase Ib/II study (NCT02331251) using gemcitabine, nab-paclitaxel, and pembrolizumab to evaluate the safety and efficacy in mPDAC improved the overall survival rate in naive chemotherapy patients (246).

A phase 1b clinical trial (NCT03307148) targeting PSCs with all-trans-retinoic-acid (ATRA) can reprogram pancreatic stroma to suppress PDAC growth (247). ATRA as a stromal-targeting agent with gemcitabine-nab-paclitaxel is safe and tolerable and will be evaluated in a phase II randomized controlled trial for locally advanced PDAC.

The clinicaltrials.gov registry provided with recent clinical trial data on PDAC having interventional therapy (**Table 2**). NCT02993731 is the largest cohort of patients with mPDAC administered nab-paclitaxel with gemcitabine. The addition of napabucasin to nab-paclitaxel with gemcitabine did not improve efficacy in patients with previously untreated mPDAC (248). CXCR2 antagonist that blocks neutrophil migration and reduces circulating neutrophil counts was studied in a clinical trial, (NCT02583477). In NCT02501902, the tolerability and antitumor activity of palbociclib plus nab-paclitaxel treatment in patients with PDAC did not meet the prespecified efficacy (249). The safety and efficacy of LMB‐100, an immunotoxin that targets mesothelin with and without nab‐paclitaxel was studied in NCT02810418 (250). This study resulted in increased numbers of active circulating CD4 and CD8 T cells, and identified specific changes in serum cytokines and peripheral CD4 T cell subsets associated with capillary leak syndrome, the major toxicity of immunotoxin therapies. NCT03611556 showed similar safety but a trend towards improved outcome (251). In NCT02289898, addition of demcizumab did not improve the efficacy in comparison with placebo (252). NCT01893801, provided encouraging results with high response rate and improved median survival (253). In NCT01658943, selumetinib plus MK-2206 did not improve overall survival in patients with mPDAC for whom gemcitabine-based chemotherapy had failed (254). The baseline immune status predicts PDAC disease course and overall survival in NCT01280058 (255). Tremelimumab monotherapy is ineffective for metastatic PDAC (NCT02527434) (256). New therapeutic options are being studied in the clinical trials: NCT02981342, NCT02558894, NCT02178709. The availability of these results and other ongoing research will help improve the future trials in PDAC patients.

**Table 2:** The clinical trial data collected from clinicaltrials.gov with keywords as pancreatic ductal adenocarcinoma and interventional study.

Study Phase	Clinical trial ID	Intervention/Treatment	Number of patients analysed	Overall survival 95% CI (Months)	Progression free survival 95% CI (Months)
Phase III	NCT02993731	Napabucasin Plus Nab-paclitaxel With Gemcitabine	565	11.43	6.70
		Nab-paclitaxel With Gemcitabine	569	11.73	6.08
Phase II	NCT02981342	Abemaciclib	33	2.71	1.68
		Abemaciclib LY3023414	33	3.29	1.81
		Gemcitabine Capecitabine	33	Data not estimable	3.25
Phase II	NCT02558894	Durvalumab (MEDI4736) monotherapy	33	3.1	1.5
		tremelimumab+MEDI4736	32	3.6	1.5
Phase II	NCT02178709	Folfirinox	43	15.7	*
Phase I- Phase II	NCT02583477	MEDI4736 in combination with nab-paclitaxel and gemcitabine	3	*	*
		MEDI4736 in combination with AZD5069	18	2.8	1.6
Phase I	NCT02501902	Palbociclib Nab-Paclitaxel	Approx 30-60 patients	**	**
Phase I- Phase II	NCT02810418	Immunotoxin (LMB-100) Nab-Paclitaxel	Approx 35-40 patients	**	**
Phase I- Phase II	NCT03611556	Oleclumab Durvalumab Gemcitabine Nab-paclitaxel Oxaliplatin Folinic acid 5-FU	213	***	***
Phase II	NCT02289898	Demcizumab Abraxane gemcitabine Placebo	207	*	*
Phase I- Phase II	NCT01893801	nab-paclitaxel Cisplatin gemcitabine	25	16.4	10.1
Phase II	NCT01658943	Akt Inhibitor MK2206 Selumetinib	58	3.9	1.9
		mFOLFOX	62	6.7	2.0
Phase II	NCT01280058	WT Reo virus Carboplatin Paclitaxel	36	7.3	4.9
		Carboplatin Paclitaxel	37	8.8	5.2
Phase II	NCT02527434	Tremelimumab monotherapy MEDI4736 monotherapy MEDI4736 + tremelimumab combination therapy	20	3.98	*

* Data not available.

** Sequential assessment.

*** Dose escalation study.

The overall survival (OS) and progression free survival of these clinical trials are given in the table.

## 9 Conclusions and perspectives

The abundant desmoplastic stroma is inextricably linked to the immune landscape of human PDAC. This dense extracellular matrix contributes to the low immunogenicity of PDAC, thereby impeding the infiltration of effector T cells and fostering an immunosuppressive TME. There exists an urgent demand to enhance our understanding of the intricate interplay among tumour cells, immune cells and stromal components within the context of pancreatic cancer. Enhancing our understanding of these interactions will be essential for improving therapeutic approaches for human PDAC.

The communication between the tumour cells and the TME is mediated by many factors including extracellular vesicles (EVs). Exosomes which are EVs with a diameter of 30-150 nm are secreted by tumour cells as well as the stromal cells in the TiME during tumour progression (257). PDAC-derived EVs distinctly regulate angiogenesis by inducing cell proliferation, mobility and secretion of pro-angiogenic factors. Cancer-associated thrombosis is yet another complication in PDAC via the expression of tissue factors. PDAC derived exosomes regulate pancreatic functions including lipidosis and glucose intake inhibition. The immunosuppressive Treg expansion is also mediated by EVs in PDAC through upregulation of the expression of FOXO transcription factors and nuclear translocation in FOXP3^+^ Tregs (258). The chemoresistant cells produce exosomal cargos that aggravate chemoresistance in sensitive cells leading to anti-apoptotic effect. Advanced PDAC patient serum has exosomes that can enhance liver and lung metastasis (259). There exists a crosstalk between tumour cells and TME mediated by EVs. PDAC-derived small EVs induce the polarisation towards M2 macrophages and inhibit effector T-cell response that promote immunosuppression and anti-tumour immunity. The potential of exosomes to stimulate the immune system of PDAC patients can be used as nanocarriers of immunotherapeutic agents (260). Therefore, understanding about this crosstalk can help develop targeted immunotherapy (261).

The immunosuppression in PDAC is multi-factorial. PDAC is characterized by an abundance of MDSCs and M2 TAMs. In contrast, the presence of CD8^+^ T cells is significantly low. The varied role of TME in PDAC can be treated by a multi-modal strategy that targets tumour promoting properties and improve the survival rate (262). The chemotherapy with immunotherapy combination tried thus far did not improve survival in mPDAC. Hence, site specific delivery of immunotherapeutics is currently under development. The ongoing clinical trials that evaluate the combination immunotherapy may elucidate mechanisms to bring down the immune suppression by TiME. The clinical trials may be evaluated further for the infiltration of adaptive immune cells like effector T cells. Patient specific biomarker identification and targeted therapy may improve the clinical outcomes. More clinical studies targeting the TiME in PDAC can enhance the potency of chemotherapy/immunotherapy treatment regimen. In general, it is thought that conceptual breakthroughs in understanding the overall TME of PDAC could facilitate the development of novel therapeutic approaches that target numerous processes simultaneously, resulting in combined benefits.

## Author contributions

AJ: Data curation, Formal analysis, Investigation, Visualization, Writing – original draft. AA: Software, Visualization, Writing – original draft, Writing – review & editing. BA-R: Funding acquisition, Writing – review & editing. SS: Conceptualization, Formal analysis, Investigation, Supervision, Writing – review & editing. UK: Conceptualization, Funding acquisition, Project administration, Supervision, Writing – review & editing.
